# Selling His Practice

**Published:** 1884-06

**Authors:** 


### Selling His Practice.

A physician sold his practice, and agreed not to practice "in that city or its vicinity." He broke this agreement, and his successor brought suit for an injunction, which was granted to him generally. The case was carried to the Supreme Court of Michigan, where the decree was confirmed. The Court defined the term "city or its vicinity" to include the territory surrounding the city for a distance of ten miles from its corporate limits. "The extent of territory, in the matter of doing business, included in the term vicinity of the city,' must necessarily depend in a great measure upon the size of the city, its location, and particular surroundings."

Dr. Philip Leidy recommends oil of citronella (two drops to the drachm) to disguise the oder of iodoform.

